# Revealing Hepatitis B Virus as a Silent Killer: A Call-to-Action for Saudi Arabia

**DOI:** 10.7759/cureus.14811

**Published:** 2021-05-02

**Authors:** Mohammed Alghamdi, Abdullah S Alghamdi, Ahmed Aljedai, Abdullah A Khathlan, Nasser A Masri, Adel Qutub, Mohammed A Quaiz, Faisal Sanai, Ghadi Subahi, Suha Sulimani

**Affiliations:** 1 Department of Gastroenterology, King Fahad Military Medical Complex, Dahran, SAU; 2 Medical Department/Gastroenterology Unit, King Fahad General Hospital, Jeddah, SAU; 3 Pharmaceutical Care Division, King Faisal Specialist Hospital and Research Centre, Riyadh, SAU; 4 Gastroenterology and Hepatology Department, King Fahad Medical City, Riyadh, SAU; 5 Department of Gastroenterology, Prince Sultan Military Medical City, Riyadh, SAU; 6 Department of Medicine, Section of Gastroenterology, King Faisal Specialist Hospital and Research Centre, Riyadh, SAU; 7 Department of Medicine, Gastroenterology Unit, King Abdulaziz Medical City, Jeddah, SAU; 8 Preventive Medicine, Ministry of Health, Riyadh, SAU; 9 Public Health, Ministry of Health, Riyadh, SAU

**Keywords:** kingdom of saudi arabia, hepatitis b virus, elimination strategy, hbv care, cirrhosis, immunization, pregnancy

## Abstract

Background: The Kingdom of Saudi Arabia (KSA) was the first country in the Middle East to adopt the hepatitis B virus (HBV) vaccine. Despite an expanded HBV immunization program and significant progress in HBV prevention in the country, HBV infection is a significant public health burden. This review lists coordinated solutions for healthcare stakeholders, patients, and health authorities to curb HBV and its impact in KSA. It further aims to draw policymakers’ attention to key priorities to bridge HBV care gaps in the country.

Methods: As part of the pre-engagement activity, medical experts across KSA were interviewed to gain a preliminary understanding of the current unmet needs in HBV management in the country. Top-recommended action points derived from the pre-engagement activity were discussed. Key priority action points to curb the impact of HBV in KSA were identified.

Results: The priority action points together with the challenges and unmet needs in the management and care of HBV in KSA were: (a) establish a national-level registry, (b) implement screening campaigns, (c) improve linkage of care between primary care physicians (PCPs) and specialists, and (d) increase PCP education and awareness.

Conclusion: This work is an endeavor to set the stage for a strategic policy framework aimed at eliminating HBV in KSA. The action points/steps for the identified priorities must run parallelly across various regions in KSA, to successfully manage and further eliminate the threat of HBV.

## Introduction

Viral hepatitis is the seventh leading cause of death in the world, with hepatitis B virus (HBV) being the most widespread viral hepatitis infection [1]. According to World Health Organization (WHO) estimates, globally in 2015, 257 million people were living with HBV infection, 71 million with hepatitis C virus (HCV) infection, and 36.7 million with human immunodeficiency virus (HIV) infection [2]. In 2015, viral hepatitis caused 1.34 million deaths worldwide, mostly from HBV infection, which is higher than the number of global deaths due to HIV infection (1.1 million) [3].

As of 2018, a whopping 290 million people worldwide, accounting for 89% of the global hepatitis B and C-infected population, were unaware that they were infected [4]. Consequently, the WHO has put forth a concrete global goal for the elimination of viral hepatitis as a public health threat by 2030. The objective is a 90% reduction in new chronic infections and a 65% reduction in deaths due to viral hepatitis [5]. This necessitates reducing the number of HBV infections to less than 1 million from 6 to 10 million infections currently, as well as reducing HBV-related deaths to 500,000 from 1.4 million by 2030 [6].

The Kingdom of Saudi Arabia (KSA) is a high-endemic country for HBV infection in the Middle East. According to the Polaris Observatory, a resource for global HBV and HCV epidemiological data, the 2016 prevalence of HBV for KSA was 2% [7]. Aljumah et al. estimated the prevalence of HBV infection in KSA to be around 1.3% [8], which is a major decline of 5-10%, compared to the figures for the 1980s [1,9]. This drop-in prevalence is primarily attributed to the introduction of HBV vaccines in 1989 for all infants at birth, and in 1990 for all school children [10,11]. The development of healthcare facilities and improved socioeconomic status have also contributed to this epidemiological decrease in HBV [9]. An epidemiological study from KSA found that the average annual incidence of seropositivity per 100,000 served population was the highest for HBV infection (104.6), followed by HCV (78.4) [10]. Indeed, HBV infection is reportedly up to 100 times more virulent than HIV infection [12]. Furthermore, considering the implications of associated comorbidities, more aggressive screening is required for HBV infection compared to HCV infection [13]. However, despite the pronounced HBV burden in KSA, HBV is not yet on the public health agenda. The transmission of HBV in KSA is predominantly horizontal through blood and its derivatives, hemodialysis, or by intravenous or percutaneous means [14]. HBV has a high rate of transplacental transmission, causing fetal and neonatal hepatitis [15]. The occurrence of HBV in KSA has declined in the past three decades, consequent to the initiation of HBV vaccination and the Ministry of Health’s (MoH) strategy for the prevention of viral hepatitis [7,16].

Similar to the global paradigm, there is a low diagnosis rate of HBV in KSA, with 2019 Polaris Observatory data indicating an HBV diagnosis rate of 7% [7]. Screening campaigns in KSA typically focus on high-risk populations, undermining the true prevalence of HBV. The incidence rate of HBV in KSA has shown an upward trend in the past five years (2014-2018) [17]. In newly diagnosed HBV cases in KSA in 2018, 14.5% of the patient pool was below 45 years of age [17]. The maximum diagnosed individuals were over 45 years of age, despite 39.2% of individuals belonging to an unspecified age group [17]. The median age of HBV patients has increased in the past five years, as has the number of comorbidities. Furthermore, the medical experts attending the workshop expect the burden of associated liver diseases to rise in the coming years, owing to aging in infected populations. The proportion of patients with hepatocellular carcinoma (HCC), cirrhosis, and hepatic steatosis has increased significantly from 2010 to 2015 [18]. Previously published evidence from KSA indicates that 40% of HCC cases in the country are due to HBV infection [14].

In 2016, HBV caused 1,700 annual deaths (i.e., 5 deaths per day) in KSA [7]. Although substantial improvements have been made in HBV management, a lot remains to be done for HBV screening and care pathways. Considering the current HBV estimates in KSA, the country is expected to achieve the WHO HBV 2030 target goals for diagnosis, treatment, and mortality by 2051 [7].

The current scenario in KSA demands the implementation of a structured policy framework to combat and eliminate HBV. Therefore, a collaborative workshop involving the Saudi Association for the Study of Liver Diseases and Transplantation (SASLT) and medical experts across KSA was convened to brainstorm on key priorities and action points for HBV management and elimination in KSA that could be highlighted to the Saudi Health Council.

The collaborative workshop was presided over by medical experts in the field of hepatology representing both the public and private sectors across KSA. The results of the collaborative workshop were detailed in a call-to-action report, which aims to summarize recommendations and key action points put forth by the medical experts to curb HBV and its impact in KSA through targeted collaborative actions.

## Materials and methods

A previous review article identified the gaps in the hepatitis B care pathway in KSA [8]. Prior to this collaborative workshop, and as part of the pre-engagement activity, IQVIA interviewed the participating medical experts through a questionnaire that gathered a 75% participation rate. The questionnaire solicited responses from the medical experts on (a) priorities in KSA that, if addressed, would have the greatest impact on a successful HBV strategy, (b) the required updates/reforms at the national level to enable the successful implementation of the above-listed priorities, and (c) the action steps that could be taken among health authorities, the medical community (healthcare professionals and patients/patient groups), payers, and others (e.g., nongovernmental organizations, laboratories, etc.) to ensure success.

**Figure 1:**
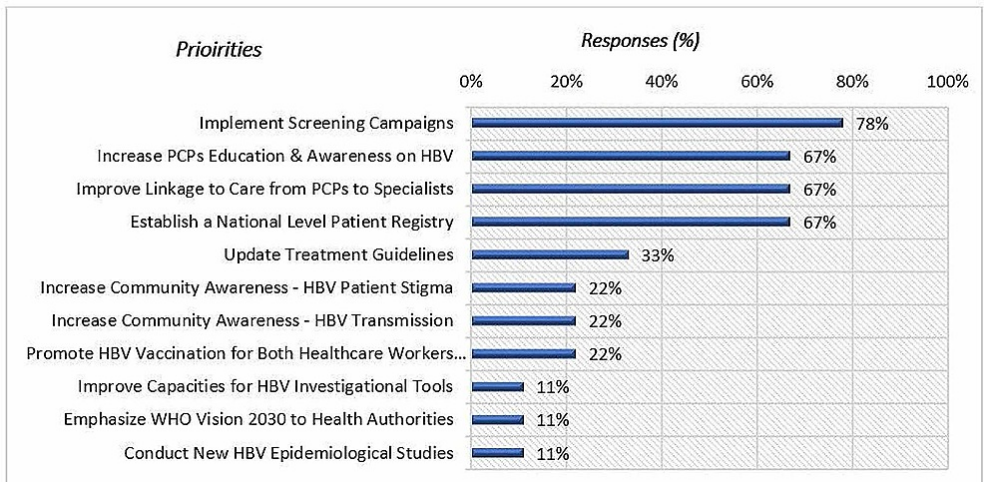
Pre-engagement survey results for priorities for combating HBV in KSA HBV: hepatitis B virus, PCPs: primary care physicians, WHO: World Health Organization.

A total of nine out of the twelve participating physicians (75%) responded to the in-depth qualitative survey. Overall, 11 priority discussion points were identified from the pre-engagement survey results. The most favored action points were as follows: implementing screening campaigns (78% response), followed by increasing primary care physicians’ (PCPs) education and awareness on HBV, improving linkage to care between PCPs and specialists, and establishing a national-level patient registry-each with 67% response (Figure 1).

The most recommended action points derived from the questionnaire results were discussed in the workshop with the medical experts, to brainstorm key priorities and action points for HBV management in KSA. Prime action points derived as a consensus from the workshop would be highlighted to the Saudi Health Council.

## Results

The available literature was reviewed to identify the burden of disease and disease management gaps for HBV in KSA. The study further identified four primary challenges or unmet needs for HBV care management in KSA.

Current situation of HBV in KSA

Consequences of HBV With Increased Age

A cross-sectional study conducted in the Aseer region highlighted the need to focus attention on the unvaccinated population, i.e., those over 25 years of age [19]. The prevalence of HBV infection in the younger population (<30 years of age) was marginal, compared to that in the older population. In a pool of 10,234 individuals, a seroprevalence of 0.8% was obtained in populations less than 15 years of age and 1.3% in the 15-24 years age group, while it stood at 6.3% in individuals aged 25 years or older [19]. In newly diagnosed HBV cases in 2018, 14.5% of the patient pool was below 45 years of age. Most diagnosed individuals (46.24%) were over 45 years of age, despite 39.2% of individuals belonging to an unspecified age group [20]. The median age of chronic HBV (CHB) patients has significantly increased from 2010 and 2015 [18].

Burden of Disease in KSA

While the prevalence of HBV disease has declined in KSA, the morbidity and mortality of HBV fail to show a similar trend. Furthermore, the burden of associated liver diseases is expected to rise in the coming years, owing to aging in the previously infected population [21]. A recent study from a primary hospital in KSA showed an increasing trend in the number of cases of primary hepatic carcinoma (PHC), which is the fourth most common malignancy among males, with the primary etiology of PHC being chronic viral hepatitis infection [22]. Poor awareness of the disease, low diagnosis rates, and treatment compliance also contribute to the disease burden. The future management of HBV patients should consider the impact of an aging HBV population with increased comorbidities and clinical outcomes such as chronic kidney disease (CKD) [2,21]. Previously published evidence from KSA indicates that 40% of HCC cases in the country are due to HBV infection [14]. The increased burden of disease in KSA highlights the importance of a targeted approach to suppress HBV endemicity in the country.

HBV Screening in KSA

HBV screening involves a blood scan to detect HBsAg, which signifies the presence of the infection. Diagnostic testing includes serology, molecular testing, liver enzyme tests, fibro scan, and transient elastography [5]. Several policies exist in KSA for HBV screening such as guidance on testing pregnant women for HBV, screening of all blood donors for hepatitis B and C, and screening of selected population groups at increased risk [23,24]. Along with the introduction of mandatory premarital HBV screening in January 2008, there are numerous potential HBV screening steps in KSA-including screening during pregnancy; premarital screening, expatriate pre-employment screening; screening of healthcare workers and medical students; and screening prior to blood donation, dialysis, and in-patient procedures [25-27].

Challenges and unmet needs in management and care of HBV in KSA

**Figure 2:**
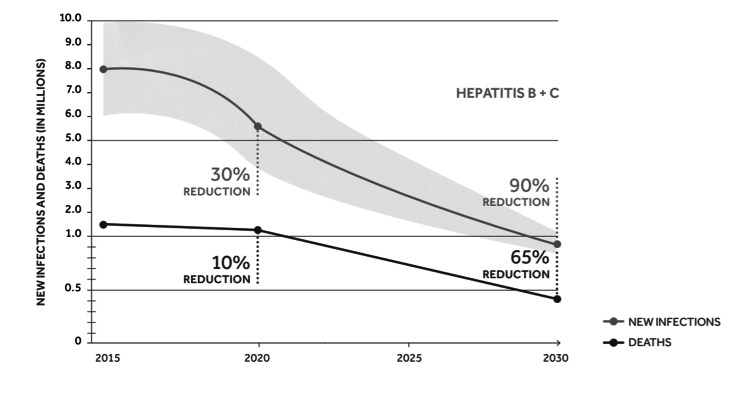
World Health Organization (WHO) 2030 goal for elimination of viral hepatitis World Health Organization, Global health sector strategy on viral hepatitis 2016-2021 [6].

The HBV burden in KSA is uncertain, as several cases go undiagnosed owing to the lack of knowledge among healthcare providers, at-risk populations, and the community at large. Despite several prevention methods and HBV testing programs being in place in the country, the coverage of screening, diagnostic testing, and treatment is still very low. According to 2019 Polaris Observatory data for KSA, the treatment rate for HBV is 4% despite an HBV vaccination rate of 98% at birth [7]. This low treatment rate can be attributed to the lack of healthcare services in less-developed regions and to the asymptomatic nature of the disease [8]. Considering the present estimates, the WHO HBV 2030 target goals for diagnosis, treatment, and mortality are expected to be achieved only by 2051 in KSA (Figure 2).

Scarcity of National-Level Patient Data

The incidence of HBV can be estimated from archived data in registries. There are limited data on the current HBV prevalence and mortality in KSA. The Systematic Observatory Liver Disease Registry is the only registry that archives data for all liver disease patients, while no exclusive registry exists for HBV patients in KSA [28].

Screening and Diagnosis

Although several policies and screening checkpoints exist in KSA for HBV, most screening programs involve targeted testing of high-risk populations. There is a limited age-stratified diagnosis for HBV. The number of reported HBV cases in the country has sustained in the last decade, despite a decline in the prevalence. The latest HBV statistics for the country exhibit a remarkably lower HBV diagnosis rate when compared to the WHO 2030 target (Table 1) [7].

**Table 1:** Comparison of WHO global HBV 2030 targets and KSA country profile HBV: hepatitis B virus; KSA: Kingdom of Saudi Arabia; PMTCT: prevention of mother-to-child transmission; PWID: persons who inject drugs.

Goals	WHO 2030 target	KSA-specific guidelines/country profile
HBV incidence	−90%	The number of patients with HBV in 2016: 532,000 [7].
HBV mortality	−65%	Annual deaths due to HBV in 2016: 1,700; 5 deaths per day [7].
HBV vaccination	90%	All healthcare facilities should abide by the HBV vaccination policy (universal neonatal or infant vaccination) [16]. In 2019, KSA achieved an HBV birth dose vaccination rate of 98% [7].
HBV PMTCT	90%	All pregnant women should be screened for HBV infection. In 2016, KSA had a 0% HBV transmission rate in pregnant women [7].
Blood and injection safety	100% screened donations, 100% safe injections	Routine serological screening of donor blood. Policy safe injection practices to prevent transmission of blood-borne infections. In 2019, KSA achieved 100% blood and injection safety [7].
Diagnosis	90%	Several potential screening steps are recommended in the Saudi population: HBV screening for pregnant women, premarital screening, expatriate pre-employment, and healthcare workers screening, pre-employment screening for military/police, screening of blood donors, in-patient screening in hospitals [11].In 2019, KSA achieved an HBV diagnosis rate of 7% [7].
Treatment	80%	In 2019, KSA achieved an HBV treatment rate of 4% [7].

PCP Education and Awareness

PCPs are usually the first point of contact for HBV diagnosis and care in KSA. Directing CHB patients to tertiary care is also supervised by PCPs. Yet not all PCPs are skilled enough to diagnose or manage HBV effectively. A cross-sectional survey of 180 primary healthcare centers in Al-Jouf Province revealed that the overall mean HBV knowledge level among physicians was 62.9%, with 43.4% of physicians being able to interpret HBV seromarkers and only 42.1% being aware of the incubation period of HBV [29]. Lack of awareness among PCPs regarding HBV is a major contributor to its under-diagnosis in the country [8]. This may be due to a lack of training programs for PCPs [29]. Training programs should therefore be encouraged among PCPs to provide increased awareness around the HBV referral chain in primary and tertiary care centers.

Linkage to HBV Care From PCP to Specialists

CHB patients diagnosed at the primary care level are sometimes directed to specialists for further treatment. In underdeveloped areas, PCPs are often required to manage CHB patients when specialists are beyond reach [8]. Monitoring of HBV diagnosed patients directed to specialized care facilities is essential. A paucity of awareness and misinterpretation of follow-up advice are root causes of poor treatment compliance among HBV patients in KSA. A study conducted in a tertiary care center involving 328 patients indicated that 40% of HBV patients were not recommended a follow-up visit, while 30% of patients were lost to follow-up [30]. Among those lost to follow-up, almost 70% were unaware that a follow-up visit had been scheduled, while 15% were not informed about a follow-up visit by the healthcare provider and 9% did not follow up due to personal beliefs. Therefore, patient counseling programs must be initiated to enhance patient compliance with HBV treatment [30].

Key priorities to combat HBV in KSA

**Figure 3:**
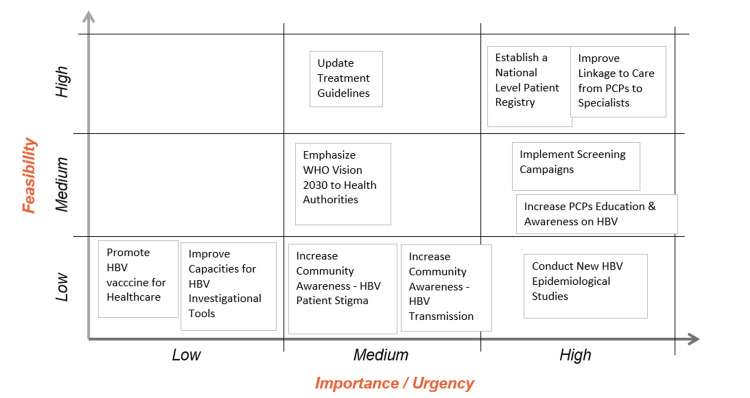
Priorities identified by the medical experts HBV: hepatitis B virus; PCPs: primary care physicians; WHO: World Health Organization.

The medical experts brainstormed and analyzed various health priorities, considering feasibility and urgency. The results are presented in Figure 3. Four high priorities were identified during the discussion and are enumerated here.

Establish a National-Level Patient Registry (Finding the Missing Numbers)

The HBV disease burden across KSA can be accurately assessed by obtaining prevalence estimates. As there are limited data on current HBV prevalence and mortality in KSA, the medical experts opined that a national-level registry should be created under the Saudi Health Council to include patients from the governmental, semi-governmental, and private sectors. This will help in generating a granular map based on the prevalence and incidence of HBV in various parts of KSA. The medical experts added that there is no need for new epidemiological studies, as a single registry will help in assessing the number of HBV-infected individuals. The national registry should be set up before further action on HBV screening campaigns, to avoid duplication of entries (for patients already diagnosed with HBV). A single national registry will not only provide data on the number of infected patients but also give insights into the demographics of the infected population. A few medical experts were in favor of conducting a burden-of-disease/cost-of-disease assessment in the country, as clinical and economic assessments would give a concrete message to policymakers on the importance of devising a structured strategy for HBV; however, this recommendation did not reach a consensus.

Implement Screening Campaigns (Identify)

Implementing an effective, targeted, customized screening program to assess the occurrence of HBV across all age groups is another key priority in the country. The vaccination program for HBV started in KSA in 1989 and reportedly reduced the prevalence to 2% [7]. However, since >95% of the young population is covered by the vaccine, the screening focus needs to shift to the adult population [8]. This includes the continuation of premarital screening and counseling of patients if found infected. In case any family member is found to be infected, the screening program should be extended to other family members as well. The experts agreed that lessons from the HCV screening campaign in KSA can be explored while devising an effective HBV screening strategy.

Improve PCP Education and Awareness (Educate and Orient)

The medical experts acknowledged the importance of PCPs and stated that they are an integral part of the management of HBV in the healthcare framework. Lack of awareness among PCPs about HBV is a major factor resulting in the under-diagnosis of HBV in the country. This can result from a shortage of primary care centers and a lack of training programs for PCPs. Therefore, educating PCPs on HBV and equipping them to ensure a timely, accurate diagnosis of the disease would increase awareness of the HBV referral chain among primary and tertiary care centers. The medical experts further added that there are about 5,000 PCPs in KSA that register HBV patient visits. Hence, educating them will require time; however, it should be done on priority. Additionally, this education initiative would, in turn, reduce the patient load among hepatologists and gastroenterologists.

Enhance Linkage to Care From PCPs to Specialists (Link)

CHB patients diagnosed at the primary care level and requiring further treatment are directed to specialists. Monitoring of HBV patients directed to specialized care facilities is essential. While PCP education is linked to enhanced diagnosis, the medical experts acknowledged the concern of patients lost to follow-up or patient unwillingness to visit a specialist due to the stigma around HBV disease. Hence, in addition to increasing patient awareness via counseling, the health system needs to be strengthened to improve the link between primary and tertiary care centers.

## Discussion

This collaborative workshop gathered recommendations and priority action points from regional medical experts to curb HBV and its impact in KSA. Four priority action points were recommended for HBV care management in KSA. For each of the priorities, specific actions are needed from health partners. While the rapidity of implementation/response in achieving key actions may vary, these action points need to be implemented in a parallel manner rather than sequentially. The key action points identified by the medical experts for moving toward a robust policy framework for the country are presented in Table 2.

**Table 2:** Action points for a robust HBV policy framework for KSA as identified by the medical experts CME: continuing medical education; HBV: hepatitis B virus; HCP: healthcare professional; KSA: Kingdom of Saudi Arabia; PCPs: primary care physicians.

Ten key action points for a robust HBV policy framework for KSA as identified by the medical experts	
1	Assessment of burden of disease and economic impact of HBV in KSA
2	Implementation of a measurable, comprehensive, and applicable referral pathway for linkage of PCPs to specialists
3	Inclusion of specific checkpoints in the referral pathway to ensure the successful patient transition
4	Use of key performance indicators to assess the referral pathway
5	Design and establishment of a tracking system in association with the MOH to monitor HBV patients (e-tracking)
6	Implementation of age-targeted screening for populations >30 years of age to assess accurate HBV prevalence
7	Customization of screening campaigns as per regional needs across KSA
8	Setting up targets and incentives for laboratories that are screening for HBV
9	Creation of free e-modules for HCPs and specifically for PCPs who may serve as first point of contact for HBV patients; with CME credits upon module completion
10	Increase of awareness for patients diagnosed with HBV via counseling sessions provided by PCPs

## Conclusions

This document aims to draw the attention of policymakers in KSA to key priorities for bridging gaps in HBV care, set the stage for "going beyond the evidence," and establish the context for a strategic policy framework for addressing HBV in KSA. The medical experts set forth KSA-specific recommendations that are complemental to the WHO 2030 target to eliminate HBV. All the recommendations consider the current evidence and practical healthcare scenario in KSA.

Implementing these strategic measures will require a multi-collaborator approach. We sincerely believe that our endeavor will provide a strong message to the Saudi Health Council regarding the need to prioritize a policy framework for the successful elimination of HBV in KSA.
